# Liquid-state carbon-13 hyperpolarization generated in an MRI system for fast imaging

**DOI:** 10.1038/ncomms14535

**Published:** 2017-03-06

**Authors:** A. B. Schmidt, S. Berner, W. Schimpf, C. Müller, T. Lickert, N. Schwaderlapp, S. Knecht, J. G. Skinner, A. Dost, P. Rovedo, J. Hennig, D. von Elverfeldt, J. -B. Hövener

**Affiliations:** 1 Department of Radiology, Medical Physics, Medical Center—University of Freiburg, Faculty of Medicine, University of Freiburg, Breisacherstrasse 60a, Freiburg 79106, Germany; 2 German Consortium for Cancer Research (DKTK), Im Neuenheimer Feld 280, Heidelberg 69120, Germany; 3 German Cancer Research Center (DKFZ), Im Neuenheimer Feld 280, Heidelberg 69120, Germany; 4 Division Hydrogen Technologies, Fraunhofer Institute for Solar Energy Systems (ISE), Heidenhofstraße 2, Freiburg 79110, Germany

## Abstract

Hyperpolarized (HP) tracers dramatically increase the sensitivity of magnetic resonance imaging (MRI) to monitor metabolism non-invasively and *in vivo*. Their production, however, requires an extra polarizing device (polarizer) whose complexity, operation and cost can exceed that of an MRI system itself. Furthermore, the lifetime of HP tracers is short and some of the enhancement is lost during transfer to the application site. Here, we present the production of HP tracers in water without an external polarizer: by Synthesis Amid the Magnet Bore, A Dramatically Enhanced Nuclear Alignment (SAMBADENA) is achieved within seconds, corresponding to a hyperpolarization of ∼20%. As transfer of the tracer is no longer required, SAMBADENA may permit a higher polarization at the time of detection at a fraction of the cost and complexity of external polarizers. This development is particularly promising in light of the recently extended portfolio of biomedically relevant *para-*hydrogen-tracers and may lead to new diagnostic applications.

## Abstract (web summary)

Hyperpolarized MRI uses molecules with a nuclear spin polarization beyond the thermodynamic equilibrium to enhance imaging contrast. Here, Schmidt *et al*. enable a single MRI system to both generate a hyperpolarized tracer and perform imaging, eliminating the need for an external polarizer.

Within the past decades, magnetic resonance imaging (MRI) has become an indispensable tool for medical diagnostics. Because of its low sensitivity, however, the only nucleus used in clinical routine is hydrogen (^1^H), which is the most abundant element *in vivo* (∼80 M) and holds the greatest gyromagnetic ratio of all stable nuclei (42 × 2*π* MHz T^−1^). But even for ^1^H, the polarization, which is the fraction of all spins contributing to the MR signal, does not exceed 3 p.p.m. in a magnetic field of 1 T. All other stable nuclei have a lower polarization and much lower abundance *in vivo*.

Still, the need for a non-invasive tool for chemical analysis *in vivo,* for example to measure the metabolism and treatment response of tumors^1^, is great. It appears that this need is precisely met by magnetic resonance (MR) spectroscopy, which is very successful for chemical analysis *in vitro*. However, low sensitivity makes its use in clinical routines very limited^2^. Nuclear hyperpolarization (HP) promises to overcome this hurdle and has enabled MR signal enhancement by several orders of magnitude^3^. The MR signal of most X-nuclei (nuclei other than protons) *in vivo* is low and thus both distribution and metabolism of magnetically labelled or hyperpolarized X-nuclei can be observed without significant background, similar to positron emission tomography. The first human studies of this method have shown very promising results^4^,^5^ and more are underway.

The most prominent techniques for the production of hyperpolarized ^13^C-tracers are dissolution dynamic nuclear polarization (dDNP)^6^ and *para*hydrogen (*p*H_2_) and synthesis allows dramatically enhanced nuclear alignment (PASADENA)^7^, also known as *p*H_2_-induced polarization (PHIP)^8^. dDNP achieves liquid-state polarization by transferring polarization from electrons to nuclei in the solid state at a temperature of a few K and in a strong magnetic field of a few T, followed by rapid dissolution. PHIP, in contrast, is the only technique to date that allows polarization to take place in the liquid state, either by hydrogenation^7^ or reversible exchange with *p*H_2_ (ref. 9). While dDNP is the only technique commercially available and used for human applications, it is associated with rather high cost, a relatively low output and long polarization cycles, although many promising attempts are being pursued to overcome these shortcomings^10^,^11^,^12^. PHIP, in contrast, is more cost efficient, very fast and requires less hardware. A hitherto substantial drawback of this technique—its limited portfolio of tracers—may have been overcome by the discovery of new agents^13^,^14^,^15^ and side-arm hydrogenation PHIP (PHIP-SAH), which has enabled the polarization of acetate and pyruvate^16^. Both methods share the disadvantage that the HP-tracer has to be produced in a dedicated polarizing device (polarizer) outside of the MRI system^3^,^6^,^17^,^18^,^19^,^20^,^21^,^22^,^23^,^24^,^25^,^26^,^27^,^28^,^29^. During the ensuing transfer through varying magnetic fields to the application site, inevitably, some of the polarization is lost to the *T*_1_ decay towards thermal equilibrium.

Here, we present a new method that achieves ^13^C-HP in excess of 20% in aqueous solution within seconds in the bore of a commercial MRI system. No external polarizer is needed—the only items that are required, in addition to an MRI scanner, are *p*H_2_, a reaction chamber, some valves and a digital output to control the process. By Synthesis Amid the Magnet Bore, A Dramatically Enhanced Nuclear Alignment (SAMBADENA) is obtained. By producing a sterile HP-tracer *in situ,* within the bore of an MRI, at the application site and at high field, this new method addresses some of the aforementioned shortcomings of dDNP and PHIP.

## Results

### SAMBADENA produces high and reproducible in-bore ^13^C-HP

**Figure 1:**
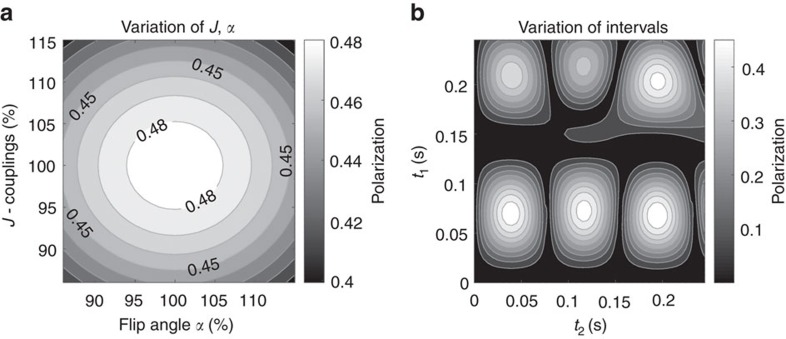
Simulated stability and intervals of l-PHINEPT for SAMBADENA. The resulting polarization of the SOT sequence l-PH-INEPT+ (longitudinal-*para*hydrogen-insensitive nuclei enhancement by polarization transfer) was simulated as function of erroneous *J*-couplings and flip angles (**a**) and as function of the evolution intervals (**b**). *J* represents the *J*-couplings of the simplified 3-spin-½ system of the tracer and *α* represents the flip angle of the pulses of the sequence; both values were varied by±15%. The values of *t*_1_ and *t*_2_ denote the free evolution intervals between the effective pulses of the SOT sequence (see Fig. 8).

A maximum theoretical polarization yield of 48.7% was simulated for 1-^13^C hydroxyenthyl-propionate (HEP), a tracer that was previously used for *in vivo* angiography^27^,^30^. The corresponding evolution intervals for the spin-order-transfer (SOT) sequence l-PH-INEPT+ (longitudinal-*para-*hydrogen-insensitive nuclei enhancement by polarization transfer) were *t*_1_=69.8 ms and *t*_2_=38.9 ms (total 108.7 ms). Several other polarization maxima were found but required longer evolution intervals (Fig. 1). The polarization yield was found to be relatively stable with respect to errors of J-couplings, pulses and evolution times (Fig. 1).

**Figure 2:**
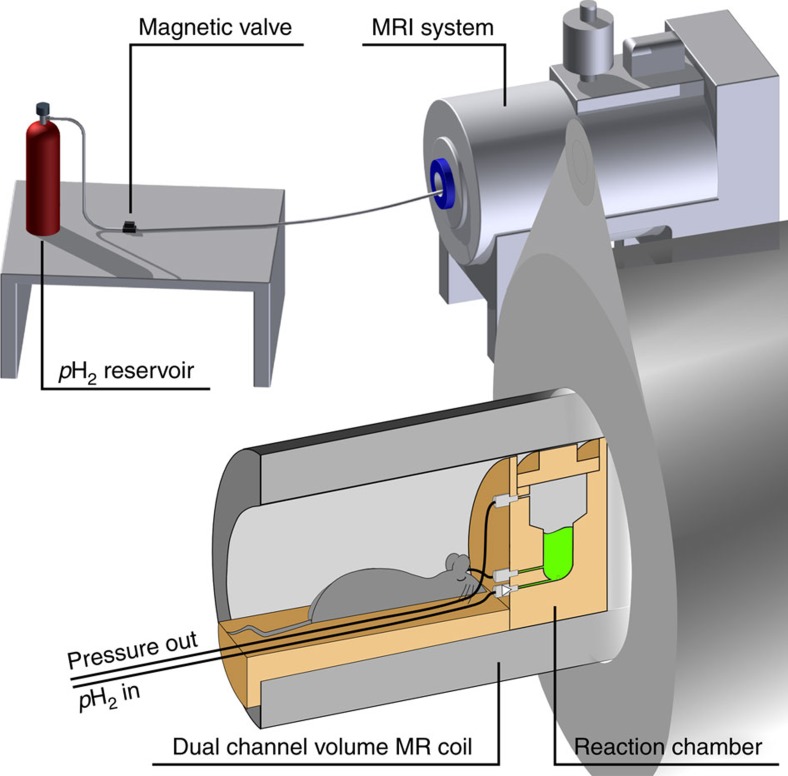
HP without a polarizer: Schematic view of the experimental setup, where the HP-tracer is produced *in situ* within the MRI close to the application site. The essential components comprise the reaction chamber, valves, tubing, *p*H_2_ and the MRI system. The latter is used to transfer the spin order and to detect the hyperpolarized signal.

**Figure 3:**
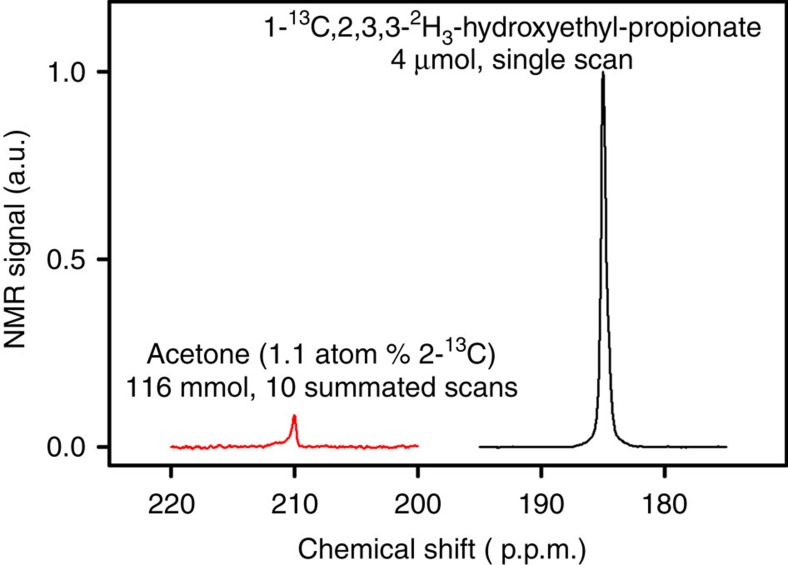
^13^C-HP generated inside an MRI system. Non-localized ^13^C-spectra of thermally polarized acetone at natural abundance (red) and ^13^C-HEP in aqueous solution (black) hyperpolarized by SAMBADENA within an MRI system. The polarization yield was quantified to ∼24%, which corresponds to a ∼40,000-fold enhancement of the thermal ^13^C-polarization which amounts to∼6 p.p.m. at 7 T.

Experimentally, HP was produced within an MRI system by using the available hardware of the system itself (Fig. 2). The signal that was directly produced by the PH-INEPT+ sequence was quantified and corresponded to a nuclear polarization of *P*=24%, approximately half of the theoretical maximum. This polarization level corresponds to an enhancement factor of *η*≈40,000 for 13C at 7 T (hydrogenation time *t*_h_=4*s*, 5.54 mM HEP in H_2_O; Fig. 3).

**Figure 4:**
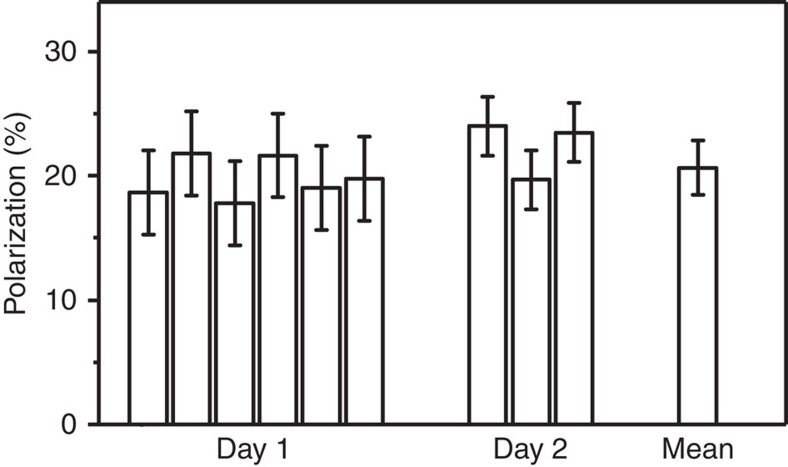
SAMBADENA reproducibility: A mean polarization of *P*=(21±2) % was achieved in nine experiments on 2 days. Error bars correspond to the s.d. of the respective day or, in case of the mean, all experiments (reaction temperature *T*≈80 °C, hydrogenation time *t*_h_=4 s, concentration of substrate *c*_HEP_=5.54 mM).

The inter-day yield and reproducibility of SAMBADENA was quantified in nine experiments to *P*≈(21±2) % with 5.54 mM HEP in H_2_O (Fig. 4).

### Production of longitudinal hyperpolarization by l-PH-INEPT+

**Figure 5:**
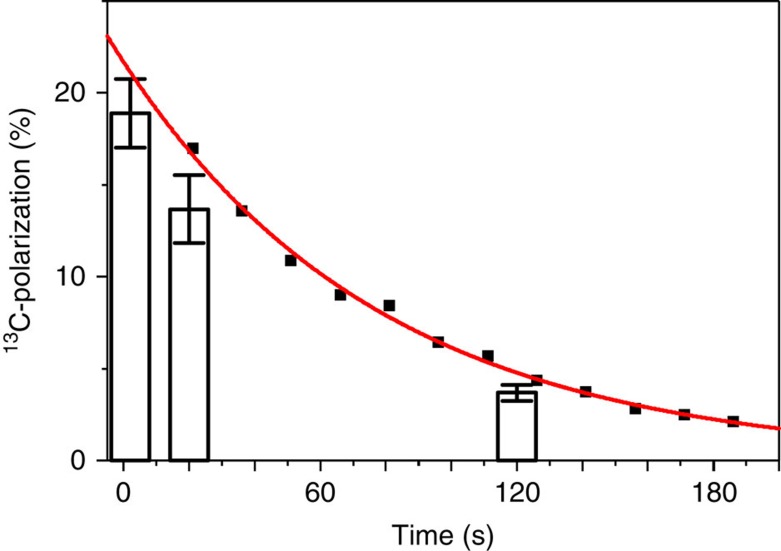
The lifetime of the polarization is long at high field. The decay of one sample of hyperpolarized ^13^C-HEP was monitored by acquiring 12 non-localized spectra with a repetition time of *T*_R_=15 s and a small flip angle of 9° (squares). A mono-exponential decay function (line) was fitted to the data and yielded *T*_1_∼(79±2) s. A correction factor—described in the methods—was applied to each data point to compensate for the fact that each measurement pulse consumes a finite amount of the magnetization. The *T*_1_ determined from the low flip angle measurement correlates well to the polarizations that were detected by full, 90° excitations 0, 20 and 120 s after the HP. For each time point, the mean of three measurements (bars) is shown, and error bars indicate the s.d. The tracer concentration was 5.54 mM in D_2_O.

While a polarization of the order of few per cent is sufficient for biomedical use^3^,^31^, the short-lived transversal polarization generated by the PH-INEPT+ sequence is not suited for *in vivo* application—longitudinal magnetization is required instead. As predicted by Bär *et al*.^32^, the l-PH-INEPT+ sequence enabled the production of longitudinal magnetization hyperpolarized to a similar level, *P*=(19±2) % (detected 2 s after generation, for 5.54 mM HEP in D_2_O) (Fig. 5). This magnetization decayed much slower with *T*_1_ (instead of *T*_2_) and enabled other experiments as described in the following.

### SAMBADENA enables magnetization storage at high field

The *T*_1_ of HEP dissolved in D_2_O was measured to (79±2) s at 7 T (Fig. 5), which is more than the 70 s reported at 4.7 T (ref. 26). In degassed and deionized H_2_O, the *T*_1_ of HEP at 7 T was measured to (75±5) s, which is less than (101±7) s reported at 48 mT (ref. 19). These long relaxation times are advantageous with respect of the further use of the tracer. If, for example, 20 s were to be used for the injection of the tracer *in vivo*, only about one fifth of the initial polarization would be lost.

### Optimized reaction conditions

**Figure 6:**
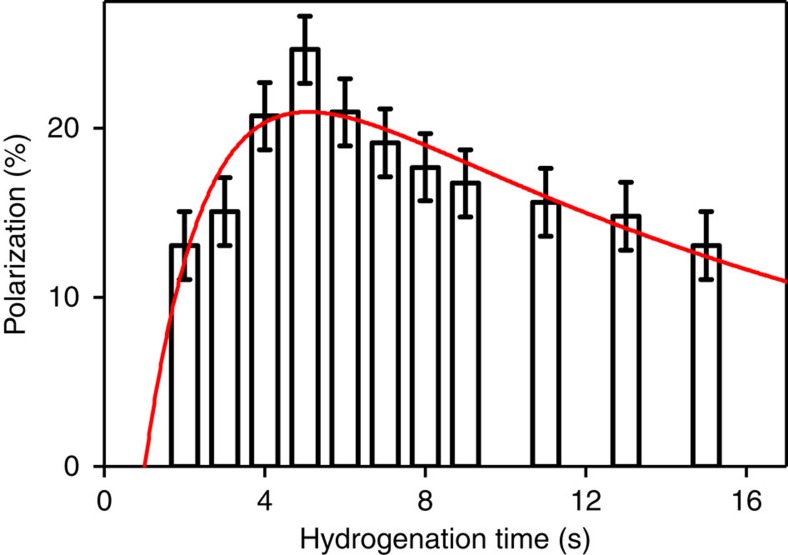
Hydrogenation time affects polarization. Mean ^13^C-HP (columns) and s.d. (error bars) of HEP (1-^13^C HEP) as a function of hydrogenation time (*t*_h_) and fit (line). In agreement with earlier reports^18^, the optimum *t*_h_ was between 4–8 s. The time constants for the catalytic hydrogenation reaction, *T*_cat_=(1.6±0.9) s, and the *para*-order relaxation, *T*_*para*_=(16±6) s, were extracted from a model fitted to the data. The model is described in Methods (equation (2)). Each time point was measured three times.

As expected and reported before^18^, the hydrogenation time had a strong impact on the HP yield: the highest polarizations were observed between *t*_h_=4–8 s (Fig. 6). Note that considerable polarization, about half of the maximum, was observed after hydrogenation periods *t*_h_ as long as 15 s. The slow decay is likely attributed to the decay of the *para*-order of the hydrogens that were added to the tracer. Note that no refocusing or decoupling pulses were applied during *t*_h_.

The apparent build-up hydrogenation time constant of the catalysis (*T*_cat_) and *para*-order relaxation constant (*T*_*para*_) were estimated to *T*_cat_=(1.6±0.9) s and *T*_*para*_=(16±6) s, respectively, by fitting a model to the experimental data (equation (2) in Methods). The onset of the hydrogenation reaction (*t*_0_) extracted from the fit was *t*_0_=(1.0±0.5) s. This value reflects the finite time required for the hydrogen being delivered to the reactor, to enter the solution, to build-up pressure and to start the reaction. It is supported by a high-speed video of the hydrogenation reaction, where the bubbling came to a stop ∼1 s after the injection, indicating an equilibration of the pressure (Supplementary Movie 1). Disregarding the contribution of relaxation, the asymptotic maximum polarization level extracted from the fit was (27±5) % (goodness of the fit: R^2^=0.7, concentrations of HEP and catalyst of *c*_HEP_=5.5 mM and *c*_cat_=2.1 mM). The deviation to the theoretical maximum may be attributed to noise in the data, relaxation before and during the SOT-sequence itself or experimental imperfections.

This data indicates that after *t*_h_=4 s, (85±18) % of all molecules are hydrogenated. Since 100% hydrogenation is assumed for the quantification of the polarization yield, it is likely that the reported polarization is underestimated. Note that *T*_cat_, *T*_*para*_, maximum polarization level and the optimal *t*_h_ are expected to be dependent on concentrations of the precursor and catalyst.

### Hyperpolarization at elevated tracer concentrations

For any biomedical application, highly concentrated, highly hyperpolarized samples are needed to achieve sufficient signal. By variation of *t*_h_, the catalyst concentration (*c*_cat_) and reaction pressure (*p*), the polarization achieved for 80 mM tracer was increased from 3.4 to 7.1% and eventually∼13% (Table 1 and Supplementary Fig. 1). It must be noted, though, that the last measurement could not be repeated because the pressure exceeded the specifications of the current instrumentation and caused damage to the equipment. In addition, *P*≈17% was readily achieved in a sample of 22 mM, 3.5 cm off the isocentre of the magnet.

### SAMBADENA and *ex vivo*^13^C-MRI

**Figure 7:**
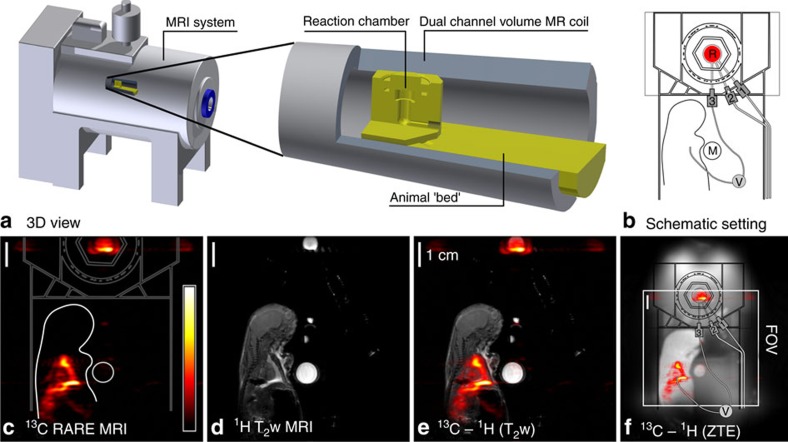
Simultaneous ^13^C-HP and *ex vivo* imaging: The compact design of the setup enabled SAMBADENA next to a small rat *post mortem* in the sensitive volume of the magnet (**a**,**b**). Within seconds, the ^13^C-tracer HEP was polarized to ∼17% at a concertation of 22 mM. Without leaving the magnet, ∼330 μl of the tracer were injected into the rat, and strong ^13^C signal with a maximum SNR of 113 was observed by MRI acquired in 0.5 s (**c**). Subsequently, a *T*_2_-weighted ^1^H-MRI (**d**) and ^1^H Zero-Echo-Time (ZTE) MRI were recorded. Co-registration of ^1^H and ^13^C-data revealed the location of the tracer, in the reaction chamber and surrounding the heart of the animal (**e**,**f**). The isocenter of the magnet is the center of the images. R, reactor; FOV, field of view; M, Model Solution M3; 1, 2, 3: lines for pressure relieve, *p*H_2_ supply and *p*H_2_ injection, respectively.

To demonstrate the feasibility of simultaneous HP and MRI, HEP was hyperpolarized by SAMBADENA within seconds, injected into a rat *post mortem* and ^13^C-MRI was acquired (*rattus rattus*, 7 days old). The rat was placed next to the reaction chamber and positioned in the sensitive volume of the coil within the magnet (Fig. 7a,b). In contrast to all other experiments, in this setting, the reaction chamber was not in the isocentre, but at a distance of 3.5 cm along the *z* axis of the magnet. At this position, the SOT sequence was still efficient, as demonstrated by an HP of 17% that was achieved for 22 mM HEP in 700 μl aqueous solution in the reactor (Table 1).

For *ex vivo* MRI, a catheter was connected to the outlet of the reaction chamber and inserted into the thorax of the rat. The HP-experiment was repeated and ∼330 μl of the hyperpolarized tracer were injected through the catheter into the rat without leaving the magnet. Sub-second ^13^C-MRI was acquired and strong, hyperpolarized signal was observed (Fig. 7c). The co-registration with a *T*_2_-weighted ^1^H-MRI (Fig. 7d,e) showed that the ^13^C-signal was localized around the heart of the animal and within the reaction chamber, with a maximum ^13^C-signal-to-noise ratio (SNR) of 113 and 111, respectively (Supplementary Fig. 2 and Supplementary Table 1). In the same scan, the SNR in the model solution was quantified to 9. A Zero-Echo-Time (ZTE) MRI^33^ was acquired afterwards to depict the entire setup (Fig. 7f).

## Discussion

SAMBADENA enables the HP of ^13^C-labelled tracers to high levels, in aqueous solution, within seconds and within the magnet bore next to the application site. For the first time, high ^13^C-HP was achieved without a dedicated polarizer—the hardware of an MRI system was used instead. This method circumvents some of the shortcomings that are associated with current ^13^C-HP methods, foremost the need of a dedicated, expensive and complex external polarizer and lengthy transfer of the sample. Other challenges persist and are discussed in the following.

Obtaining a pure solution devoid of the catalyst is a persisting challenge for all *p*H_2_-based HP methods, although strong progress was reported recently by using either heterogeneous catalysts^34^,^35^,^36^,^37^ or a biphasic approach^38^. The latter appears to be particularly promising with respect to the implementation in the presented setup, and heterogeneous catalysts are currently being investigated in our laboratory.

Whereas other HP methods require an extra polarizer device whose complexity, operation and cost can exceed that of the MRI scanner itself, the additional equipment needed here is very little. The only extra component that is needed inside the magnet is a reaction chamber. In the experiments presented here, the bulk reactor occupies less than one-third of the imaging field of view (FOV). A significant reduction in size appears feasible because the actual inner volume of the reactor is less than 1/100 of the FOV (2 cm^3^ versus 342 cm^3^). The only requirement for the MRI system is a ^13^C channel, which is necessary for ^13^C-MRI in general.

A relatively large volume transmitter is advantageous to enable simultaneous application of the SOT and MRI sequence. A dedicated pickup coil at the region of interest (ROI) may assure a high sensitivity while the homogeneous excitation of the large volume coil (covering both chamber and animal) would be maintained. To improve the accuracy of the applied pulse sequences, two different shim settings were used; one for the HP and one for the imaging.

SAMBADENA may be simplified further by using the digital outputs of the MRI system to control the valves; its implementation on any commercial multinuclear MRI system appears feasible. Other *p*H_2_-based approaches are much less cost intensive than dDNP, but still require an external polarizer device with the associated cost and complexity^17^,^18^,^19^,^21^,^22^,^23^,^26^,^27^,^29^,^29^.

While the actual HP process is executed with a single button, the production of *p*H_2_ and precursor solution requires some training. In the future, both *p*H_2_ and solution may be prepared elsewhere and shipped to the application site. At this stage, the catalyst–substrate solution is filled manually into the reactor outside the scanner. We envision that future implementations will feature in-bore loading, automatic heating and likely automated injection^18^.

Any preparation of the hyperpolarized tracer, such as cooling or quality control, would take place at the elevated field of the host MRI system where the *T*_1_ is long (here:∼80 s). Note, however, that any human application would require fast and comprehensive testing of the solution before injection, to ensure the safety of the patient, which is challenging especially in the magnet bore.

The HP levels reached −∼20% for a tracer concentration of 6 and 22 mM, and 13% for 80 mM—are likely sufficient for a meaningful *in vivo* application, in particular because no lengthy transfer of the tracer through varying magnetic fields is necessary.

The SOT-sequence l-PH-INEPT+ was implemented for the first time, and about half of the theoretically achievable polarization yield was observed (*P*=(19±2) %). Other sequences with a higher theoretical yield appear feasible and may improve the polarization as well.

Fast hydrogenation at elevated temperature and high pressure was found to be essential for a high polarization (Fig. 2 and Supplementary Fig. 1). The catalytic hydrogenation time constant extracted from experimental data indicated that (85±18) % of a 5.5 mM precursor solution were hydrogenated after a 4 s reaction at 15 bar and∼80 °C. Especially with respect to a high substrate concentration, an even higher pressure and temperature is expected to improve the yield further (see Supplementary Fig. 1).

Polarization losses may be attributed to relaxation before, during and after the polarization transfer, imperfect pulses, timings (or erroneous *J*-coupling constants) and incomplete hydrogenation. We observed that the reaction chamber distorted the line shape of a model solution, which may also cause polarization loss (see Supplementary Figs 3 and 4). Note that 100% hydrogenation and *p*H_2_ enrichment was assumed for the quantification, which results in some underestimation of the reported polarization.

The technical feasibility of using the MRI simultaneously for the HP and subsequent imaging was demonstrated by an *ex vivo* experiment (Fig. 7). Although there appears to be no fundamental hurdle for a preclinical *in vivo* application, the following aspects have to be taken into account: First, the equipment to support the animal (anaesthesia, vital sign monitoring, heating) have to fit into the bore along with the animal and the reactor. This issue may be addressed by employing a smaller reactor designed to be used with a commercial animal bed. Second, the injection needs to be well controlled regarding flow, pressure and total volume. An automated injection system for dDNP that was already described and evaluated positively in many *in vivo* experiments^39^ may be used here. Third, unwanted heating of the animal (by the reactor) should be avoided, and the HP-solution needs to be cooled to temperature that is acceptable for injection. This first implementation was not designed with an *in vivo* demonstration in mind, but the temperature at the position of the animal is close to the required range (measured as 42 to 37 °C as a function of distance from the outside wall of the reactor, see Supplementary Fig. 5). The temperature at the position of the animal may be reduced further by adding insulation and/or using a different method of heating. As for the temperature of the solution, an elevated temperature is required only at the time of the hydrogenation (to provide fast catalysis, a high polarization yield and sterilize the tracer solution, see Supplementary Note 1). The temperature of the tracer exiting the catheter used *in vitro* was measured to (43±3)°C and was further reduced to (32±3)°C by guiding the catheter through iced water (Supplementary Table 2). Thus, it appears feasible to reach an acceptable temperature within seconds. Current work focuses on adapting SAMBADENA for the HP of biomolecules to conduct metabolic studies *in vivo*.

A major limitation of *p*H_2_-based methods was the hitherto restricted portfolio of relevant tracers. However, this hurdle may have been overcome by the HP of pyruvate and acetate^16^, phospholactate^13^,^14^,^15^, and (diethyl-) succinate^1^,^40^,^41^,^42^,^43^ with *p*H_2_. Current work focuses on adapting SAMBADENA to these tracers.

Compared with other methods, the HP of a liquid-state tracer next to the application site may enable the delivery of higher polarization *in vivo* at the time of detection, especially for agents with short relaxation time *T*_1_ (such as glucose), and enable better resolution of metabolites with hitherto low SNR.

Given its simplicity, its high polarization yield, fast production and low cost, SAMBADENA may have an important impact on the research of HP tracers, which hold the potential to revolutionize the use of MRI in modern diagnostics.

## Methods

### *Para*hydrogen production

*p*H_2_ was enriched to ∼95% similar as descried previously and stored in aluminium bottle to be used on demand^44^.

### Experimental setup

Measurements were performed using a 7 T preclinical small animal MRI system (Biospec 7/20, PV5.1, Bruker, Germany), using a dual-resonant ^1^H–^13^C transmit-receive volume coil (Rapid, Germany; Fig. 2).

A reaction chamber with an inner volume of ∼2 ml was custom-made from polysulfone (PSU 1000) to allow a high hydrogenation temperature (∼80 °C) and pressure (∼15 bar). Tubes were connected to the inlet at the bottom (1/8” × 1/16”, PTFE, SCP GmbH, Germany) and outlet at the top (1/16” × 0.75 mm, PTFE, SCP GmbH, Germany) to inject and release *p*H_2_, respectively. Both tubes were connected to magnetic valves (type 0124, Bürkert, Germany) outside of the MR system. The valves were controlled by a custom-written software (MATLAB, MathWorks, USA) using the digital outputs of a data acquisition board (DAQ 6125, National Instruments, USA). A second reactor was used for hydrogenation experiments (Fig. 6) and *ex vivo* MRI experiments, where an additional outlet at the bottom served to inject the tracer into the animal (Fig. 7).

### Preparation of the samples

A rhodium-based catalyst was prepared fresh by mixing a biphosphine ligand (1,4-bis-[(phenyl-3-propane sulfonate) phosphine] butane disodium salt, Q36333, MW=562.53 g mol^−1^, Sigma Aldrich, MO, USA) with a rhodium complex (bis(norbornadiene)rhodium (I) tetrafluoroborate, MW=373.99 g mol^−1^, CAS 36620-11-8, StremChemicals, MA, USA) in degassed D_2_O (Deuterium oxide, 99.9 atom % D, CAS 7789-20-0, Sigma Aldrich, MO, USA) or degassed and deionized H_2_O (ref. 17).

In contrast to previous reports^17^,^26^, the rhodium moiety was dissolved in warm H_2_O/D_2_O (∼60 °C) instead of acetone.

**Figure 8:**
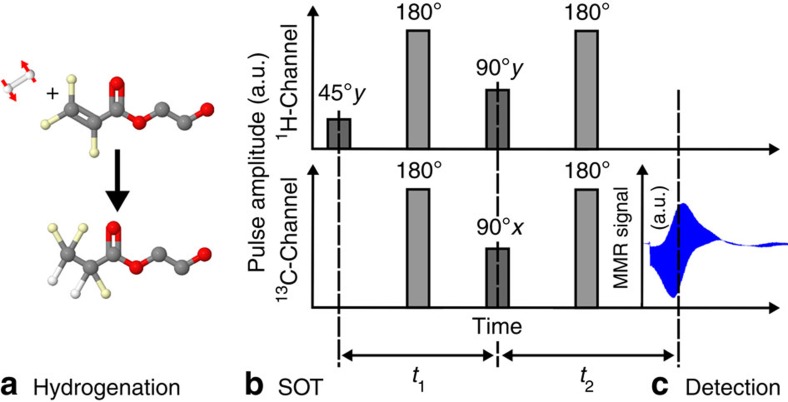
Schematic view of the HP method: The tracer to-be-hyperpolarized is formed by catalytic addition of *p*H_2_ to an unsaturated precursor molecule (**a**, here: hydroxyethyl-acrylate forms the angiography-tracer^27^,^30^ HEP). Next, a pulse sequence is applied subsequently to transfer the spin order (grey bars, here: PH-INEPT+ (ref. 45)), and the enhanced ^13^C signal is recorded (**c**). In **b**, refocusing pulses are shown in light grey. (**c**) Real ^13^C-data in the time domain acquired after one HP experiment.

1-^13^C, 2,3,3-^2^H_3_-hydroxyethyl-acrylate (HEA, 99% ^13^C, MW=120.13 g mol^−1^, CAS: 1216933-17-3, Sigma Aldrich, USA) was added to the solution. During the HP experiment, *p*H_2_ was added to HEA and 1-^13^C, 2,3,3-^2^H_3_-HEP was formed (Fig. 8).

To achieve a high hydrogenation temperature, but to avoid degeneration of the catalyst^18^, a stock solution containing catalyst and substrate at high concentrations was prepared and kept at room temperature (concentrations: *c*_rhodium_=21 mM, *c*_ligand_=23 mM, *c*_HEA_=55.4 mM). The reaction chamber was heated in a water bath to ∼90–95 °C. Shortly before the experiment, one part (100 μl) of the stock and nine parts of hot H_2_O or D_2_O (∼90 °C) were filled into the reaction chamber and placed in the MRI scanner. Unless otherwise indicated, the final concentration was 2.1 mM for the catalyst (with 10% excess of the ligand) and 5.54 mM of HEA.

For the experiment with elevated tracer concentrations, the required amount of HEA was added to the reactor afterwards (for example 9 mg to reach 80 mM in 1 ml solvent).

This procedure permitted obtaining a high hydrogenation temperature of >80 °C, while keeping the catalyst at room temperature for most of the time^18^.

### Spin order transfer

Both PH-INEPT+ (ref. 45) and l-PH-INEPT+ (ref. 31) sequences were implemented on the MRI system. The PH-INEPT+ sequence, which consists of three excitation pulses (45°y-^1^H, 90°y-^1^H and 90°x-^13^C) interleaved by two refocusing pulses (180° on ^1^H and ^13^C), was used to transform the *para*-spin order into hyperpolarized transversal ^13^C magnetization (Fig. 8). The transversal magnetization generated by this method is short-lived and decays with *T*_2_*. The l-PH-INEPT+ (ref. 31) sequence, instead, generates long-lasting longitudinal magnetization by the addition of an extra 90°y-^13^C pulse to PH-INEPT+. Note that the evolution intervals between the pulses of the sequences (*t*_1_ and *t*_2_) depend on the hydrogen-hydrogen- and hydrogen-carbon *J*-couplings of the target molecule.

Quantum mechanical simulations were carried out to optimize *t*_1_ and *t*_2_ and to determine the theoretical polarization yield. The simulations were performed using the product operator and density matrix formalism in a reduced spin system containing the two *para*-hydrogens and the target nucleus ^13^C (*J*-couplings: *J*_H-H_=7.57 Hz, *J*_H1-C_=7.24 Hz *J*_H2-C_=−5.62 Hz)^46^. It was assumed that only longitudinal spin order survives the hydrogenation process, and that evolution takes place under the isotropic liquid-state Hamiltonian. The optimal timings with respect to the highest polarization yield were obtained by numerical optimization. Relaxation was neglected and rotation operators were applied to mimic the effect of radio frequency pulses. To assess the effect of erroneous intervals, flip angles and *J*-couplings, these parameters were varied^31^,^47^.

### MR settings

For *in vitro* experiments, 1 ml of H_2_O was filled into the reactor and placed in the isocentre of the scanner (referred to as position 1) to match and tune the coil and to adjust the frequency and field homogeneity (iterative first-order shim). The ^1^H frequency was manually set to ∼2.8 p.p.m. The ^13^C frequency was set to ∼185 p.p.m., the chemical shift of 1-^13^C HEP, using the ^13^CO-resonance of acetone (referred to as model solution M1, 1.1 atom % natural abundance of carbon-13, 8 ml, concentration of ∼14 M, chemical shift of the ^13^CO resonance 210 p.p.m.).

The ^13^C flip angle was calibrated manually using free induction decays with varying pulse power and an 8 ml sample of ethanol at natural abundance (reference pulse gains for a 1 ms, 90° pulse of 53.6 W (17.2 dB) for ^1^H and 163.5 W (8.2 dB) for ^13^C).

For the imaging experiments, the reactor was placed at a distance of 3.5 cm to the isocentre (referred to as position 2). The ^1^H and ^13^C flip angles were calibrated using a model solution containing 667 mg 1-^13^C sodium acetate in 2 ml deionized H_2_O (denoted as model solution M2, longitudinal relaxation time *T*_1_=(22±1) s (see Supplementary Fig. 6), 99 atom % ^13^C, MW=83.03 g mol^−1^, *c*=4 M, CAS 23424-28-4, Sigma Aldrich, USA) in a cutoff 10 mm nuclear magnetic resonance (NMR) tube that was placed within the reaction chamber. Compared with the flip angles measured at position 1, deviations of 1% for ^1^H and 18% for ^13^C were found (reference pulse gains of 54.2 W (17.1 dB) for ^1^H and 190.5 W (7.1 dB) for ^13^C (Supplementary Fig. 7).

Before HP, the first-order shims and frequencies were adjusted for the reactor and the rat using a PRESS^48^ localization. Both settings were saved and loaded before HP or imaging was conducted.

The receiver gain was set to 64 dB for all ^13^C-experiments.

### Hyperpolarization

After the reactor was filled with the precursor solution, closed and placed in the magnet, the automated HP procedure was started, consisting of the following steps: First, the hydrogenation reaction was initialized by injecting *p*H_2_ at 15 bar (unless indicated otherwise) for a period of 2 s, followed by a variable delay of 1–12 s, yielding a total hydrogenation time of *t*_h_=3–14 s. Next, the SOT-sequence was executed (∼100 ms). If the PH-INEPT+ sequence was used, the ^13^C signal was detected directly at the end of *t*_2_; if l-PH-INEPT+ was applied, the magnetization was read out after another delay *t*_d_, either by non-localized spectroscopy or imaging. Note that ∼300 μl of the solution was flushed into the outlet tube during the hydrogenation.

### Longitudinal relaxation time *T*_1_

After an l-PH-INEPT+ experiment, the *T*_1_ of HEP in D_2_O and H_2_O was measured by probing the decaying longitudinal magnetization with small-angle acquisitions of 9°, interleaved by 15 s (D_2_O) or 5 s (H_2_O). *T*_1_ was extracted from an exponential decay function *S*(*t*)=*S*_0_·exp(−*t*/*T*_1_)·cos(*α*)^*n*−1^, where *S(t)* is the acquired signal, *S*_0_ is the signal at *t*=0, *α* the flip angle (9°) and *n* the number of the pulse of the series. The absolute polarization of this scan was estimated using the signal of the first acquisition and *α*. Note, that the data points in Fig. 5 were corrected for polarization loss induced by the previous excitations by dividing the measured signals by cos(*α*)^*n*−*1*^.

### Quantification of HP

The absolute polarization yield *P* was quantified with respect to the signal of a thermally polarized sample (M1) for the *in vitro* experiments, and a model solution containing 333 mg 1-^13^C sodium acetate dissolved in 1.2 ml deionized H_2_O (denoted as model solution M3, concentration of sodium acetate of *c*=3.3 M) for the *ex vivo* experiments:


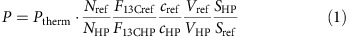


where HP and ref indicate a value of the hyperpolarized sample and the reference model solution, *N* is the number of summated scans (1 for all HP experiments), *F*_13C_ is the atomic fraction of the ^13^C isotope, *c* is the concentration of the molecule, *V* is the volume of the sample, *S* the measured ^13^C-NMR signal and *α* the excitation flip angle, *P*_therm_ is the thermal ^13^C-polarization at 7T and room temperature (∼6 p.p.m.). Note that 100% *p*H_2_ enrichment and complete hydrogenation was assumed, which results in an underestimation of the HP that was actually achieved.

The NMR signals were numerically integrated after multiplication with an exponential function (10 Hz), Fourier-transformation and automated phase and baseline correction (Topspin 2.0, Bruker, Germany).

### Hydrogenation

The quantification of the hydrogenation yield by NMR of the samples after HP is problematic because the hydrogenation reaction continues after HP. Indeed, complete conversion was found in all samples investigated. Attempts to monitor the conversion during the reaction failed because of low signal (localized spectroscopy, PRESS at 9.4T and∼100 mM substrate concentration) or strong distortions of the resonances (non-localized spectroscopy).

Instead, the HP yield (*P*) was recorded as a function of the hydrogenation time *t*_h_ at otherwise identical conditions. From the resulting data, an apparent *para*-order relaxation time *T*_*para*_ and a hydrogenation constant *T*_cat_ were obtained: we hypothesize that *P* is governed by a saturated growth described by *T*_cat_ and a mono-exponential decay of the *para*-order (*T*_*para*_) after the hydrogenation occurred. By solving the differential equations describing the chemical kinetics, an analytical expression for *P*(*t*_h_) was derived and fitted to the experimental data. The function *P*(*t*_h_) reads:


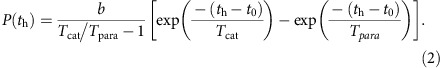


The coefficients *b* and *t*_0_ allow the maximum polarization level to be adjusted and a time delay between the application of *p*H_2_ pressure and the onset of the hydrogenation reaction, respectively.

### *Ex vivo* experiments

An *ex vivo* rat (*rattus rattus*, 35 g, 7 cm, 7 days old) and model solution M3 were placed next to the reaction chamber. An injection hose (42 cm, 1/16'', PTFE, SCP GmbH, Germany) was connected to the outlet of the reactor and inserted into the thorax of the rat. The setup was positioned in the coil within the MRI system. The reactor was at a distance of 3.5 cm from the isocentre (position 2). The field homogeneity was optimized for two volumes, the position of the reactor and the entire rat. The settings were saved and loaded when needed. After the HP, *p*H_2_ pressure was released, and a manual valve (V in Fig. 7) was actuated to inject the HP-tracer directly into the rat without leaving the magnet.

Two-dimensional single-shot RARE (rapid acquisition with relaxation enhancement)^49^ images were acquired before, as well as∼10 s and ∼41 s after the HP was generated (90/180°, RARE factor 38, partial Fourier factor 1.7778, 38·96 matrix, FOV (8.4 cm)^2^, zero-filled to 128·128, (0.65 mm)^2^ in-plane resolution, 6-cm-slice thickness, acquisition time/*T*_R_=0.487 s, *T*_E_=79 ms, acquisition time 487 ms, bandwidth 10 kHz, centred in the isocentre).

Subsequently, a *T*_2_-weighted, two-dimensional, ^1^H-Turbo-RARE sequence was acquired with the same in-plane FOV for anatomical reference (90/180°, RARE factor 8, bandwidth 46.9 kHz, matrix 256^2^, in-plane resolution (328 μm)^2^, *T*_R_=2.5 s, *T*_E_=33 ms, acquisition time 80 s, 15 slices 1 mm thick, 1.5 mm apart, centred in the isocenter).

A ZTE MRI was acquired to display the reaction chamber and rat simultaneously (2.3°, matrix 128^3^, FOV 16.8 cm, *T*_R_=4 ms, acquisition time 208 s, bandwidth 300 kHz, 51,896 projections, centred in the isocentre).

The SNR was calculated by dividing the maximum signal intensity of a chosen ROI by the standard deviation of the noise (paravision 5.1, Bruker, Germany) (Supplementary Fig. 2 and Supplementary Table 1). For the co-registration, black was set transparent in the ^13^C image (Fig. 7) (GNU image manipulation program). The ZTE data are shown as a max. intensity projection (imageJ^50^). The images were composed with Inkscape.

### Data availability

The authors declare that all relevant data presented here are available from the corresponding author upon reasonable request.

## Additional information

**How to cite this article:** Schmidt, A. B. *et al*. Liquid-state carbon-13 hyperpolarization generated in an MRI system for fast imaging. *Nat. Commun.* **8,** 14535 doi: 10.1038/ncomms14535 (2017).

**Publisher's note:** Springer Nature remains neutral with regard to jurisdictional claims in published maps and institutional affiliations.

## Supplementary Material

Supplementary Information Supplementary Figures, Supplementary Tables, Supplementary Note and Supplementary Reference.

Supplementary Movie 1 The video shows the injection of *para*hydrogen (*p*H_2_) into the reaction chamber at a pressure of 15 bar, and temperature of ~ 20°C. The video was recorded at 240 frames per second corresponding to a slow-motion factor of 10. The reactor was filled with 1 mL of H_2_O.

## Figures and Tables

**Table 1:** Experimental parameters and corresponding polarization yields.

***P*** **(%)**	***p*****(*****p*****H**_**2**_**) (bar)**	***c***_**cat**_ **(mM)**	***c***_**HEP**_ **(mM)**	***t***_**h**_ **(s)**	**Solvent**
3.4	15	2.1	80	4	D_2_O
7.1	15	2.1	80	8	D_2_O
13	30	4.2	80	8	H_2_O
17	15	4.2	22	5	H_2_O

The hyperpolarization of HEP (^13^C-hydroxyethyl-propionate) at a concentration of *c*_HEP_=80 mM was increased by variation of the *para-*hydrogen pressure (*p*(*p*H_2_)), the concentrations of the catalyst (*c*_cat_) and the hydrogenation time (*t*_h_).

## References

[b1] Kurhanewicz J. . Analysis of cancer metabolism by imaging hyperpolarized nuclei: prospects for translation to clinical research. Neoplasia 13, 81–97 (2011). 21403835 10.1593/neo.101102 PMC3033588

[b2] Xu V. . MR spectroscopy in diagnosis and neurological decision-making. Semin. Neurol. 28, 407–422 (2008). 18843570 10.1055/s-0028-1083685

[b3] Ardenkjaer-Larsen J. H. . Dynamic nuclear polarization polarizer for sterile use intent. NMR Biomed. 24, 927–932 (2011). 21416540 10.1002/nbm.1682

[b4] Nelson S. J. . Metabolic imaging of patients with prostate cancer using hyperpolarized [1-13C]pyruvate. Sci. Transl. Med. 5, 108 (2013). 10.1126/scitranslmed.3006070 PMC4201045 23946197

[b5] Cunningham C. H. . Hyperpolarized 13C metabolic MRI of the human heart—initial experience. Circ. Res. 119, 1177–1182 (2016). 27635086 10.1161/CIRCRESAHA.116.309769 PMC5102279

[b6] Ardenkjær-Larsen J. H. . Increase in signal-to-noise ratio of >10,000 times in liquid-state NMR. Proc. Natl Acad. Sci. USA 100, 10158–10163 (2003). 12930897 10.1073/pnas.1733835100 PMC193532

[b7] Bowers C. R. & Weitekamp D. P. Parahydrogen and synthesis allow dramatically enhanced nuclear alignment. J. Am. Chem. Soc. 109, 5541–5542 (1987).

[b8] Eisenschmid T. C. . Para hydrogen induced polarization in hydrogenation reactions. J. Am. Chem. Soc. 109, 8089–8091 (1987).

[b9] Adams R. W. . Reversible interactions with para-hydrogen enhance NMR sensitivity by polarization transfer. Science 323, 1708–1711 (2009). 19325111 10.1126/science.1168877

[b10] Comment A. . Producing over 100 ml of highly concentrated hyperpolarized solution by means of dissolution DNP. J. Magn. Reson. 194, 152–155 (2008). 18595751 10.1016/j.jmr.2008.06.003 PMC2575746

[b11] Batel M. . A multi-sample 94 GHz dissolution dynamic-nuclear-polarization system. J. Magn. Reson. 214, 166–174 (2012). 22142831 10.1016/j.jmr.2011.11.002

[b12] Bornet A. . Boosting dissolution dynamic nuclear polarization by cross polarization. J. Phys. Chem. Lett. 4, 111–114 (2013). 26291221 10.1021/jz301781t

[b13] Shchepin R. V., Coffey A. M., Waddell K. W. & Chekmenev E. Y. PASADENA Hyperpolarized 13C Phospholactate. J. Am. Chem. Soc. 134, 3957–3960 (2012). 22352377 10.1021/ja210639c PMC3318994

[b14] Shchepin R. V., Pham W. & Chekmenev E. Y. Dephosphorylation and biodistribution of 1-13C-phospholactate *in vivo*. J. Label. Compd. Radiopharm. 57, 517–524 (2014). 10.1002/jlcr.3207 PMC4287379 24995802

[b15] Shchepin R. V., Coffey A. M., Waddell K. W. & Chekmenev E. Y. Parahydrogen induced polarization of 1-13C-phospholactate-d2 for biomedical imaging with >30,000,000-fold NMR Signal Enhancement in Water. Anal. Chem. 86, 5601–5605 (2014). 24738968 10.1021/ac500952z PMC4063326

[b16] Reineri F., Boi T. & Aime S. ParaHydrogen Induced Polarization of 13C carboxylate resonance in acetate and pyruvate. Nat. Commun. 6, 5858 (2015). 25556844 10.1038/ncomms6858

[b17] Hövener J.-B. . PASADENA hyperpolarization of 13C biomolecules: equipment design and installation. Magn. Reson. Mater. Phys. 22, 111–121 (2009). 10.1007/s10334-008-0155-x PMC2664858 19067008

[b18] Kadlecek S. . A simple and low-cost device for generating hyperpolarized contrast agents using parahydrogen. NMR Biomed. 24, 33–42 (2011). 10.1002/nbm.1757 21845739

[b19] Waddell K. W., Coffey A. M. & Chekmenev E. Y. *In situ* detection of PHIP at 48 mT: demonstration using a centrally controlled polarizer. J. Am. Chem. Soc. 133, 97–101 (2011). 21141960 10.1021/ja108529m PMC3098502

[b20] Wagner, S.. Agraz J. Li D., Devices and methods for parahydrogen induced polarization. US patent 2,217,262 (2015).

[b21] Agraz J., Grunfeld A. M., Cunningham K., Li D. & Wagner S. Improved PHIP polarization using a precision, low noise, voltage controlled current source. J. Magn. Reson. 77–84 (2013). 10.1016/j.jmr.2013.08.001 23988431

[b22] Agraz J. . LabVIEW-based control software for para-hydrogen induced polarization instrumentation. Rev. Sci. Instrum. 85, 044705 (2014). 24784636 10.1063/1.4870797

[b23] Agraz J. . PHIP instrumentation pinch valve system for sample delivery, process and collection. Adv. Biomed. Sci. Eng. 1, 8–18 (2014).

[b24] Comment A. . Principles of operation of a DNP prepolarizer coupled to a rodent MRI scanner. Appl. Magn. Reson. 34, 313–319 (2008).

[b25] Jannin S. . A 140 GHz prepolarizer for dissolution dynamic nuclear polarization. J. Chem. Phys. 128, 241102 (2008). 18601309 10.1063/1.2951994

[b26] Hövener J.-B. . Quality assurance of PASADENA hyperpolarization for 13C biomolecules. Magn. Reson. Mater. Phys. 22, 123–134 (2009). 10.1007/s10334-008-0154-y PMC2664864 19067009

[b27] Goldman M., Johannesson H., Axelsson O. & Karlsson M. Design and implementation of C-13 hyperpolarization from para-hydrogen, for new MRI contrast agents. Comptes Rendus Chim. 9, 357–363 (2006).

[b28] Johannesson H., Axelsson O. & Karlsson M. Transfer of para-hydrogen spin order into polarization by diabatic field cycling. C R Phys. 5, 315–324 (2004).

[b29] Coffey A. M. . Open-source automated parahydrogen hyperpolarizer for molecular imaging using 13C metabolic contrast agents. Anal. Chem. 88, 8279–8288 (2016). 27478927 10.1021/acs.analchem.6b02130 PMC4991553

[b30] Olsson L. E. . MR coronary angiography in pigs with intraarterial injections of a hyperpolarized 13C substance. Magn. Reson. Med. 55, 731–737 (2006). 16538605 10.1002/mrm.20847

[b31] Dutta P., Martinez G. V. & Gillies R. J. A new horizon of DNP technology: application to *in-vivo* 13C magnetic resonance spectroscopy and imaging. Biophys. Rev 5, 271–281 (2013). 26491489 10.1007/s12551-012-0099-2 PMC4610403

[b32] Bär S. . On the spin order transfer from parahydrogen to another nucleus. J. Magn. Reson. 225, 25–35 (2012). 23103392 10.1016/j.jmr.2012.08.016

[b33] Weiger M., Pruessmann K. P. & Hennel F. MRI with zero echo time: hard versus sweep pulse excitation. Magn. Reson. Med. 66, 379–389 (2011). 21381099 10.1002/mrm.22799

[b34] Koptyug I. V. . Para-hydrogen-induced polarization in heterogeneous hydrogenation reactions. J. Am. Chem. Soc. 129, 5580–5586 (2007). 17408268 10.1021/ja068653o

[b35] Kovtunov K. V., Beck I. E., Bukhtiyarov V. I. & Koptyug I. V. Observation of parahydrogen-induced polarization in heterogeneous hydrogenation on supported metal catalysts. Angew Chem. Int. Ed. Engl. 47, 1492–1495 (2008). 18205153 10.1002/anie.200704881

[b36] Kovtunov K. V., Zhivonitko V. V., Corma A. & Koptyug I. V. Parahydrogen-induced polarization in heterogeneous hydrogenations catalyzed by an immobilized Au(III) complex. J. Phys. Chem. Lett. 1, 1705–1708 (2010).

[b37] Glöggler S. . A nanoparticle catalyst for heterogeneous phase para-hydrogen-induced polarization in water. Angew. Chem. Int. Ed. Engl. 54, 2452–2456 (2015). 25565403 10.1002/anie.201409027

[b38] Reineri F. . Use of labile precursors for the generation of hyperpolarized molecules from hydrogenation with parahydrogen and aqueous-phase extraction. Angew Chem. Int. Ed. Engl. 50, 7350–7353 (2011). 21698723 10.1002/anie.201101359

[b39] Comment A. & Van Der Klink J. Infusion pump with phase separator. US patent 8,034,027 (2011).

[b40] Chekmenev E. Y. . PASADENA hyperpolarization of succinic acid for MRI and NMR spectroscopy. J. Am. Chem. Soc. 130, 4212–4213 (2008). 18335934 10.1021/ja7101218 PMC2662769

[b41] Bhattacharya P. . Towards hyperpolarized 13C-succinate imaging of brain cancer. J. Magn. Reson. 186, 150–155 (2007). 17303454 10.1016/j.jmr.2007.01.017 PMC2657725

[b42] Ross B. D., Bhattacharya P., Wagner S., Tran T. & Sailasuta N. Hyperpolarized MR imaging: neurologic applications of hyperpolarized metabolism. Am. J. Neuroradiol. 31, 24–33 (2010). 19875468 10.3174/ajnr.A1790 PMC7964072

[b43] Zacharias N. M., Chan H. R., Sailasuta N., Ross B. D. & Bhattacharya P. Real-time molecular imaging of tricarboxylic acid cycle metabolism *in vivo* by hyperpolarized 1-13C diethyl succinate. J. Am. Chem. Soc. 134, 934–943 (2012). 22146049 10.1021/ja2040865 PMC3262122

[b44] Hoevener J.-B. . A continuous-flow, high-throughput, high-pressure parahydrogen converter for hyperpolarization in a clinical setting. NMR Biomed. 26, 124–131 (2013). 22833391 10.1002/nbm.2827

[b45] Haake M., Natterer J. & Bargon J. Efficient NMR pulse sequences to transfer the parahydrogen-induced polarization to hetero nuclei. J. Am. Chem. Soc. 118, 88–91 (1996).

[b46] Goldman M. & Johannesson H. Conversion of a proton pair para order into 13C polarization by rf irradiation, for use in MRI. Comptes Rendus Phys. 6, 575–581 (2005).

[b47] Hövener J.-B., Knecht S., Schwaderlapp N., Hennig J. & von Elverfeldt D. Continuous re-hyperpolarization of nuclear spins using parahydrogen: theory and experiment. Chem. Phys. Chem. 15, 2451–2457 (2014). 25079961 10.1002/cphc.201402177

[b48] Bottomley P. A. Spatial localization in NMR spectroscopy *in vivo*. Ann. N. Y. Acad. Sci. 508, 333–348 (1987). 3326459 10.1111/j.1749-6632.1987.tb32915.x

[b49] Hennig J., Nauerth A. & Friedburg H. RARE imaging: a fast imaging method for clinical MR. Magn. Reson. Med. 3, 823–833 (1986). 3821461 10.1002/mrm.1910030602

[b50] Schneider C. A., Rasband W. S. & Eliceiri K. W. NIH Image to ImageJ: 25 years of image analysis. Nat. Methods 9, 671–675 (2012). 22930834 10.1038/nmeth.2089 PMC5554542

