# Supplementary material for: Liquid-state carbon-13 hyperpolarization generated in an MRI system for fast imaging
Source: Nat Commun. 2017 Mar 6;8:14535. doi: 10.1038/ncomms14535 (PMC5343473; doi:10.1038/ncomms14535)
Supplement: Supplementary Information — Supplementary Figures, Supplementary Tables, Supplementary Note and Supplementary Reference. [file ncomms14535-s1.pdf]

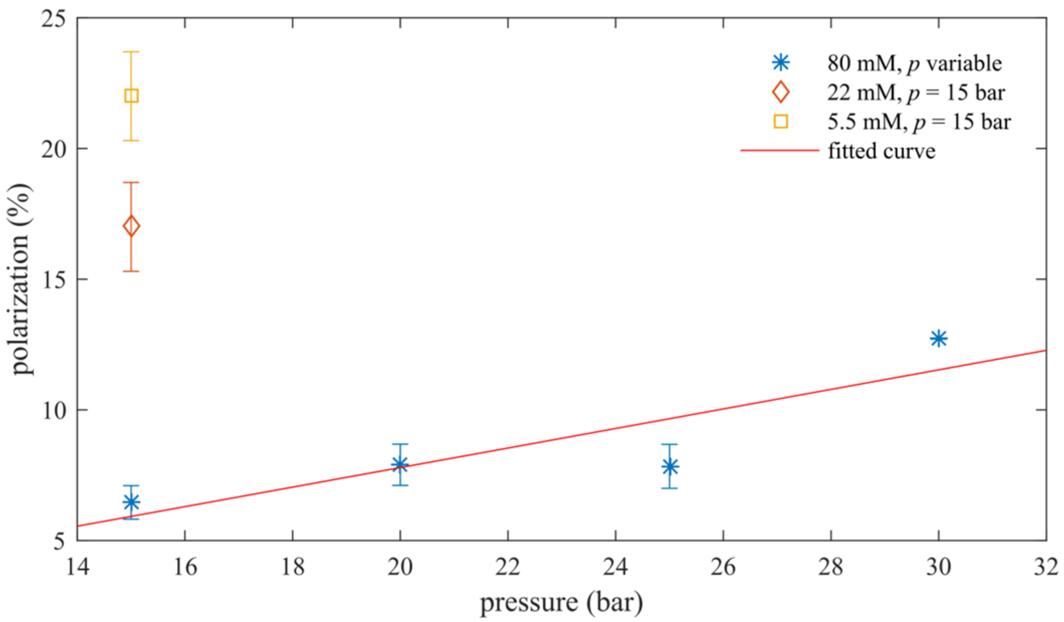

Supplementary Figure 1: ^{13}C-hyperpolarization yield as function of pressure and tracer concentration. Several HP experiments with different tracer concentrations were performed using the equipment and quantification as described in the main manuscript. For 5.5 mM tracer concentration, $P \approx 22\%$ was observed (15 bar, 4 s hydrogenation, 2.1 mM catalyst concentration, 1 mL, squares). For 22 mM tracer concentration, $P \approx 17$ % was observed (15 bar, 5 s hydrogenation, 4.2 mM catalyst concentration, 1 mL, diamonds). For 80 mM tracer concentration, the polarization yield increased with the applied pH_2 pressure (p) to a maximum of 13 % at 30 bar (asterisks).

A linear function was fitted to the 80 mM data and yielded the following parameters: $P(p) = (0.37 {*} p \text{ bar}^{-1} + 0.32)$ % (goodness of fit: adjusted $R^2 = 0.66$, hydrogenation time: 8s, catalyst concentration 4.2 mM in 1 mL H_2O, two hyperpolarization experiments for 15 bar to 25 bar, one for 30 bar, Matlab 2015b, the Mathworks, USA). The error bars indicate the standard deviation of the mean values.

Unfortunately, the valves controlling the pH_2 supply and pressure outlet did not withstand the elevated pressure, which is why experiments had to be stopped after one measurement at 30 bar. Still, the hyperpolarization yield achieved appears suitable for *in vivo* use. There appears to be much room to improve the polarization yield with respect to tracer concentration, catalyst concentration, pressure, hydrogenation duration and temperature.

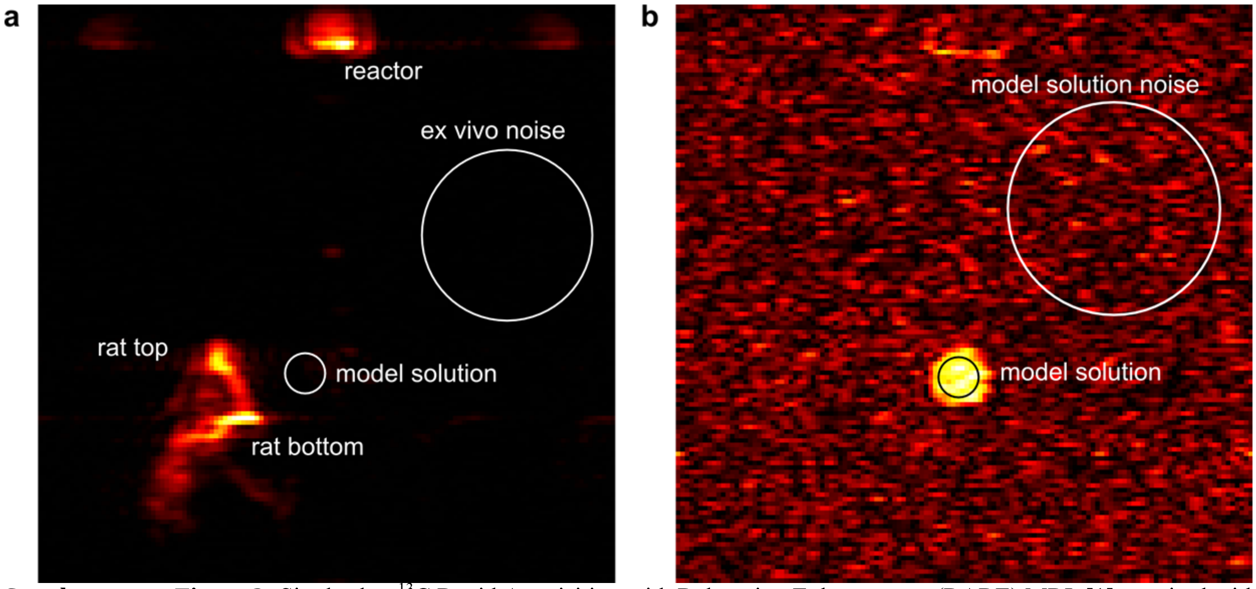

Supplementary Figure 2: Single shot ^{13}C Rapid Acquisition with Relaxation Enhancement (RARE) MRI [1] acquired within 2 s (a) and 31 s after (b) the hyperpolarized tracer was injected into a rat *post mortem* using an excitation flip angle of $\alpha = 90$ °. The regions that were used to calculate the signal to noise ratios (SNR) using the manufacturers software are indicated. The MRI was acquired with a partial Fourier factor set to 1.77778, which corresponds to 96/1.7778 = 54 (=48 + 6) *k*-space lines. However, the sequence was implemented by the manufacturer such that only 38 lines were acquired, which corresponds to the RARE factor. Thus, the size of the data that was in fact acquired was 96 × 38 (corresponding to a nominal resolution of 0.875 × 2.22 mm^2). This data was zero-filled to 128 × 128 for the signal to noise ratio (SNR) measurement (resolution (656 µm)^2). To facilitate the coregistration with the ^{1}H MRI, the data was zero-filled to the matrix size of the ^{1}H image (256 × 256). No significant difference between the 128^2 and 256^2 images was visible to the naked eye. The color-scheme used was “red hot” (imageJ) and black was set transparent for the overlay (www.gimp.org).

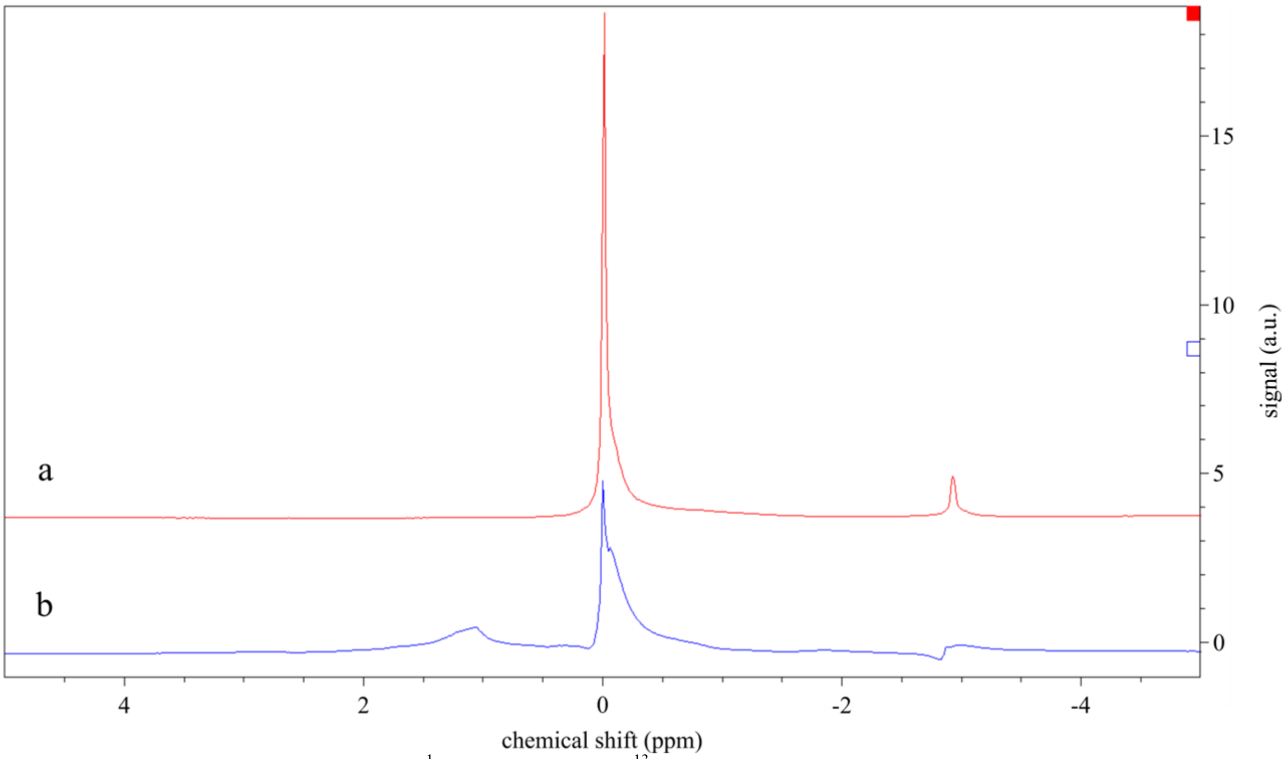

Supplementary Figure 3: Non-localized ^{1}H-spectra of 4 M 1-^{13}C sodium acetate dissolved in 2 mL H_2O (denoted as model solution M2 in the manuscript) that was placed in the reaction chamber (b) or in a polystyrene holder (a) (flip angle 10°, single scan, 0.5 ms pulse length, 12800 Hz excitation bandwidth). When the model solution was placed in the reaction chamber used for hyperpolarization, a distorted line shape was observed in the ^{1}H spectra which was not be improved by shimming (b). No distortions were observed when the model solution was placed in a polystyrene holder (a). The origin of the distortions is not yet known. It may be due to the equipment (reaction chamber, fittings, tubings, etc.), or to depositions in the reaction chamber. It is possible that the effect on the hyperpolarized tracer is stronger than on the phantom (which was held in a shortened 10 mm nuclear magnetic resonance tube), because the tracer is in direct contact with the reaction chamber. The inhomogeneities may cause a reduced accuracy of the spin-order transfer sequence or accelerated relaxation.

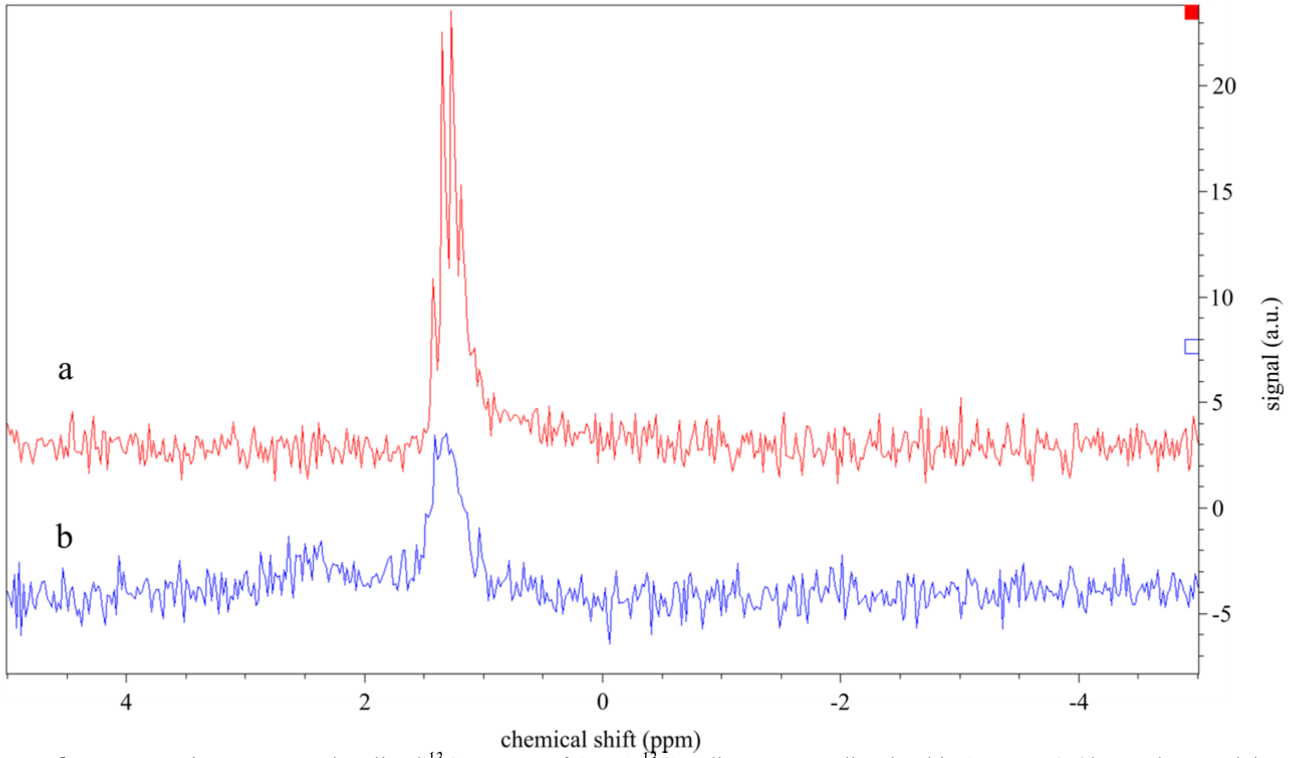

Supplementary Figure 4: Non-localized ^{13}C-spectra of 4 M 1-^{13}C sodium acetate dissolved in 2 mL H_2O (denoted as model solution M2 in the manuscript) that was placed in the reaction chamber (b) or in a polystyrene holder (a) (flip angle 10°, number of acquisitions $N = 20$, 0.5 ms pulse length, 2560 Hz excitation bandwidth). When the model solution was placed in the reaction chamber used for hyperpolarization, a distorted line shape was observed in the ^{13}C spectra which was not be improved by shimming (b). No distortions were observed when the model solution was placed in a polystyrene holder (a). The origin of the distortions is not yet known. It may be due to the equipment (reaction chamber, fittings, tubings, etc.), or to depositions in the reaction chamber. It is possible that the effect on the hyperpolarized tracer is stronger than on the phantom (which was held in a shortened 10 mm nuclear magnetic resonance tube), because the tracer is in direct contact with the reaction chamber. The inhomogeneities may cause a reduced accuracy of the spin-order transfer sequence or accelerated relaxation.

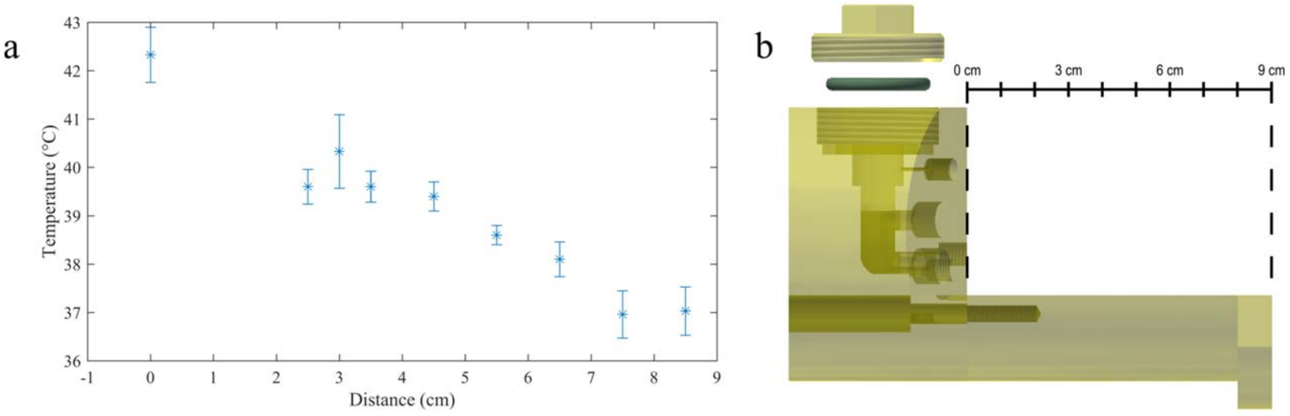

Supplementary Figure 5: Temperature measured at a given distance from the heated reactor (a) and schematic diagram of the reactor (b). Each data point (asterisks) is the mean of three measurements and the error bars are the corresponding standard deviations. For the *ex vivo* experiment, the rat was located between 0.5 cm and 7 cm from the reactor wall (at 0 cm, (b)), where the temperatures were in between 38 °C to 42 °C. Prior to every temperature measurement, the reaction chamber was placed in hot water (~ 95 °C) for several minutes. Next, the reactor was taken out of the water bath, and the temperature of the polysulfone attachment of the reactor (where the rat was placed later) was measured after 10 s for different distances from the reactor using a fast temperature sensor (Voltcraft 502, K/J – TypeDual Input Thermometer, Voltcraft, CH). The temperature is close to values acceptable for *in vivo* experiments. A different method of heating and an insulation layer are expected to lower the temperature further.

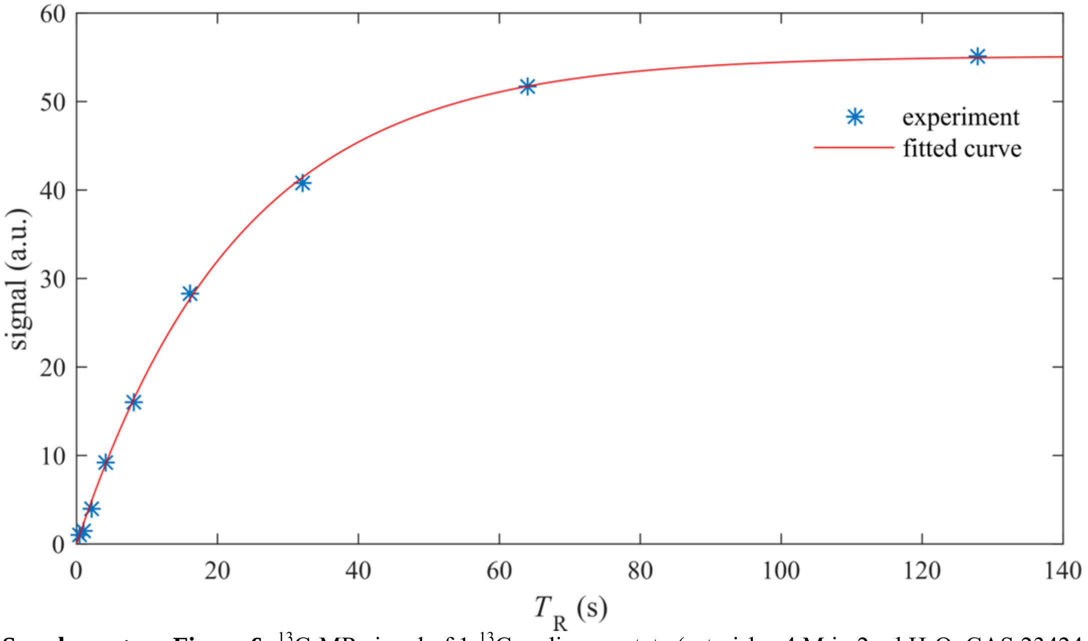

Supplementary Figure 6: ^{13}C-MR signal of 1-^{13}C-sodium acetate (asterisks, 4 M in 2 ml H_2O, CAS 23424-28-4, Sigma Aldrich, USA) acquired with non-localized spectroscopy as function of repetition time (T_R) and saturation recovery fit (line). Each data point corresponds to the summated signal of 20 acquisitions. The signal intensities were quantified by numerical integration after multiplication with an exponential function (10 Hz), Fourier-transformation and automated phase and baseline correction (Topspin 2.0, Bruker, Germany).

A saturation recovery function, $S(T_R) = a \times (1 - e^{-T_R/T_1}) + c$, was fitted to the data and a longitudinal relaxation time of T_1 = (22 ± 1) s was extracted (adjusted R^2 = 0.99, Matlab 2015b, the Mathworks, USA).

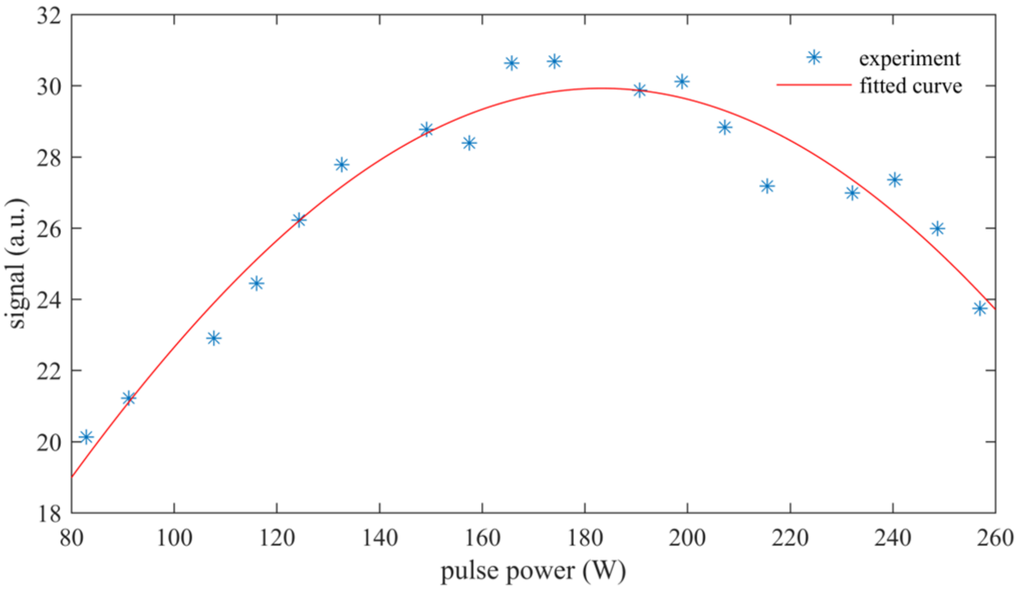

Supplementary Figure 7: ^{13}C signal of 1-^{13}C-sodium acetate (asterisks, 4 M in 2 ml H_2O, CAS 23424-28-4, Sigma Aldrich, USA) at a distance of 3.5 cm from the isocenter of the magnet along the z-axis acquired with non-localized spectroscopy for different pulse powers (pulse width = 1 ms, repetition time T_R = 120 s ≈ 5·T_1, N = 20 for each power setting) and fit (line).
A sine function $f(x) = a \cdot \sin(b \cdot x + c)$ was fitted to the data, and the following parameters were extracted: a = (29.9 ± 0.3), b = (0.0086 ± 0.0003), c = (0.003 ± 0.05), goodness of fit: adjusted R^2 = 0.94, Matlab, the Mathworks, USA).The resulting reference power was 190.5 W (6.8 dB) when the reactor was at a distance of 3.5 cm from the isocenter in z-direction, compared to 161 W (8.195 dB) at the isocenter ((which one is) not shown).

Scan No.	N	ROI	Mean (a.u.)	SD (a.u.)	Max. (a.u.)	Size (voxels)	SNR
**1 (135)**	**3**	**Noise**	7.81×10^4	4.04×10^4	2.68×10^5	3004	
		**M3**	6.21×10^5	8.52×10^4	7.48×10^5	61	**18.5**
**2 (142)**	**1**	**Noise**	4.87×10^4	3.61×10^4	2.18×10^5	1032	**-**
		**Rat Bottom**	2.52×10^6	9.23×10^5	4.11×10^6	30	**113**
		**Rat Top**	2.06×10^6	5.08×10^5	2.83×10^6	30	**78**
		**Reactor**	2.04×10^6	9.2×10^5	4.02×10^6	35	**111**
		**M3**	1.97×10^5	6.13×10^4	3.27×10^5	61	**9**
**3 (143)**	**1**	**Noise**	4.5×10^4	2.45×10^4	1.46×10^5	1384	
		**M3**	2.28×10^5	2.97×10^4	3.08×10^5	86	**13**

Supplementary Table 1: Evaluation of ^{13}C MRI. Quantified mean signal, standard deviation, maximum and size of the region of interest (ROI) of an MRI acquired before the injection (scan 1), within two seconds after the injection (scan 2), and 31 s after the injection (scan 3). Identical parameters were used for all sequences as described in the main manuscript, with the exception of scan 1, which was acquired three times (N) with T_R = 2 m.

	**Temperature of water (°C)**	
**Measurement No**	setting 1 (air cooling)	setting 2 (iced water)
**1**	40	35
**2**	45	30
**3**	46	31
**4**	43	
**Mean**	43	32
**SD**	3	3

Supplementary Table 2: To measure the temperature of the hyperpolarized tracer, a syringe was filled with 1 mL of H_2O and placed in an 80 °C water bath for > 5 min. From this syringe, 500 µL H_2O were injected within 2 seconds into a vial (Eppendorf Safe-Lock Tube) through a catheter (1/16'', PTFE, SCP GmbH, Germany) of 42 cm length (incl. fittings and a shut-off valve). The temperature in the receiver was measured using a sensor (Voltcraft 502, K/J – TypeDual Input Thermometer, Voltcraft, CH) placed in the ejected stream of the catheter for two configurations: either the catheter was in contact with air at ambient temperature (~ 23°C, denoted as setting 1) or the catheter was guided through iced water (~ 0 °C, denoted as setting 2). The experiments were repeated several times. The mean and standard deviation were calculated to (43 ± 3) °C for setting 1 (ambient air cooling), and to (32±3) °C for setting 2 (iced water). These results demonstrate that it is feasible to produce a hyperpolarized tracer solution at a temperature that is acceptable for *in vivo* experiments within seconds.

Department für Medizinische Mikrobiologie und Hygiene
Medizinaluntersuchungsamt für Freiburg und den
Landkreis Breisgau-Hochschwarzwald

Institut für Medizinische Mikrobiologie und Hygiene
Hermann-Herder-Straße 11
79104 Freiburg i. Br.
Tel.: 0761/203-6510
Ärztlicher Direktor: Prof. Dr. med. G. Häcker

UNIVERSITÄTS KLINIKUM FREIBURG

Institut f. Med. Mikrobiologie u. Hygiene, Postfach 820, 79008 Freiburg

Universitätsklinikum Freiburg
Hauspost

Patient: Steri-Test 1
geb.: Pat.-Nr.: X1254549

Endbefund
Auftrag 1254549 vom 07.10.2016

Material: Sonstiges -1- / in Blutkulturflasche (aerob)

Abnahme: 07.10.2016
ca. 0,20 ml in BK-Flaschen
Tagesnummer: ster85213
Externe ID: ARHWYY9J
Befunddatum: 25.10.2016

Kein Wachstum von Bakterien/Pilzen
Die Steriltestung erfolgte mit dem automatisierten Nachweisverfahren BacT/ALERT 3d (Biomerieux)in den Flaschentypen FA Plus. Bebrütungszeit 16d/ Bebrütungstemperatur 35°C.

Material: Sonstiges -1- / in Blutkulturflasche (anaerob)

Abnahme: 07.10.2016
ca. 0,20 ml in BK-Flaschen
Tagesnummer: ster85214
Externe ID: NRH869V2
Befunddatum: 25.10.2016

Kein Wachstum von Bakterien/Pilzen
Die Steriltestung erfolgte mit dem automatisierten Nachweisverfahren BacT/ALERT 3d (Biomerieux)in den Flaschentypen FN Plus. Bebrütungszeit 16d/ Bebrütungstemperatur 35°C.

Material: Sonstiges -2-

Abnahme: 07.10.2016
Gesamtmenge ca. 0,3ml
Tagesnummer: ster85215
Befunddatum: 25.10.2016

Kein Wachstum von Bakterien/Pilzen
Die Steriltestung erfolgte durch Anlage des Materials auf den festen Nährmedien Columbiablut-Agar (Oxoid) in aerober Atmosphäre und Hefe-Cystein-Blut-Agar (HCB; eigene Herstellung) in anaerober Atmosphäre und einem flüssigen

Test 1

Material: **-1- / Blood culture flask (aerobic)**
Sample date: 7th of October, 2016
Volume 0.20 ml
Date of inspection: 25th of October, 2016

Result: No growth of bacteria/fungi
Detection method: BacT/ALERT 3d (bioMérieux, France) in FA Plus bottles
Incubation temperature 35°C
The cultures are incubated for 16 days (ending on 23rd of October 2016).

Test 2

Material **-1- / Blood culture flask (anaerobic)**
Sample date: 7th of October, 2016
Volume 0.20 ml
Date of inspection: 25th of October, 2016

Result: No growth of bacteria/fungi
Detection method: BacT/ALERT 3d (bioMérieux, France) in FN Plus bottles
Incubation temperature 35°C
The cultures are incubated for 16 days (ending on 23rd of October 2016).

Test 3

Material **-2-**
Sample Date: 7th of October, 2016
Volume 0.3 ml
Date of inspection: 25th of October, 2016

Result: No growth of bacteria/fungi
The sterility test was performed by testing the growth on solid columbia agar media in aerobic atmosphere and yeast cysteine blood agar in aerobic atmosphere and in a liquid brain-heart-infusion broth.
Incubation temperature 36°C
Incubation time 7 days.

Supplementary Note 1: Sterility of the produced HP solution was tested by the Institute for Microbiology and Hygiene of the University Medical Center Freiburg. An English transcript is provided.

Supplementary References

[1] J. Hennig, A. Nauerth, H. Friedburg, RARE imaging: A fast imaging method for clinical MR, Magn. Reson. Med. 3 (1986) 823–833. doi:10.1002/mrm.1910030602.
